# By the Way

**Published:** 1913-01-11

**Authors:** 


### BY THE WAY.

### SURGEON AND PATIENT IN ONE.

With regard to patients operating on themselves Dr. Etienne Mieulet reports in *Paris Médical* a case in which a patient removed a stone from his own bladder. Sub-Assistant-Surgeon-Major Clever, of the Garde Royale, having had no less than five operations for the removal of stones from the bladder, was suffering from his sixth attack of vesical pain. Worn out with the agony caused by the new calculus by which he had been tormented for a year, during a paroxysm of pain he had the courage himself to remove the cause of his troubles. The following is the account of his operation which he communicated to the *Journal Médico-Chirurgical du Var* for 1824: "Firm in my intention, after making the necessary preparations, I placed myself in the proper position before a looking-glass, lifted the scrotum with my left hand which at the same time made the skin of the perineum tense, and then plunged the point of a bistoury into the region where the operation is usually performed until it met the stone which was lying at the neck of the bladder. Having made this puncture, I incised the skin and introduced a finger into the wound, thinking thus to reach the foreign body, but the point of the bistoury having only cut by slipping towards the interior the division was imperfect. After a moment's rest I placed my instrument again in the wound and completed the incision. Then with the help first of one finger and next of two together, the index and middle, I sought for and soon succeeded in extracting a stone the size of a large nut. On completing the operation urine flowed out in abundance; I dressed the wound with compresses soaked in an emollient decoction, and, quite relieved, went to bed and slept soundly. The following day I was as calm and cheerful as though I had never suffered. Several doctors of my acquaintance, friends and colleagues, as well as a large number of persons with whom I was acquainted, surprised by this news, flocked to my house to make certain of a fact which to them seemed astonishing. Professor Béclard himself visited me and examined the stone." From that moment M. Clever made a rapid and complete recovery.

